# Incidence of Autism Spectrum Disorder in Youths Affected by Gilles de la Tourette Syndrome Based on Data from a Large Single Italian Clinical Cohort

**DOI:** 10.3390/brainsci10110812

**Published:** 2020-11-02

**Authors:** Mariangela Gulisano, Rita Barone, Maria Rita Mosa, Maria Chiara Milana, Federica Saia, Miriam Scerbo, Renata Rizzo

**Affiliations:** Child and Adolescent Neurology and Psychiatric Section, Department of Clinical and Experimental Medicine, Catania University, 95124 Catania, Italy; rbarone@unict.it (R.B.); mariaritamosa@libero.it (M.R.M.); mariachiara.milana@gmail.com (M.C.M.); federicasaia@live.com (F.S.); mimiscerbo@gmail.com (M.S.); rerizzo@unict.it (R.R.)

**Keywords:** Gilles de la Tourette syndrome, autism spectrum disorder, comorbidity, incidence

## Abstract

Gilles de la Tourette syndrome (GTS) and autism spectrum disorder (ASD) are etiologically related neurodevelopmental disorders with an onset age before 18 years and a reported comorbidity of 2.9–20%. The aim of the present study was to identify the incidence of ASD in a large clinical sample of individuals affected by GTS and to compare our results with previously reported incidences. We retrospectively analyzed clinical data (*n* = 1200) from January 2010 to March 2019 obtained from the outpatient Catania Tourette Clinic, part of the Child and Adolescent Neurology and Psychiatry of the Medical and Experimental Department of Catania University. We used internationally validated evaluation tools. The neuropsychological evaluation was carried out by an expert and a certificated team of child and adolescent neurologists, supervised by two expert child neurologists (R.R. and M.G.). We investigated 975 GTS-affected individuals of various socioeconomic levels aged 5–18 years, and 8.9% (*n* = 87) were affected by ASD. The incidence of GTS with ASD was significantly lower (*p* < 0.001) in children than in adolescents. No statistically significant differences were found in the sex distribution and age of onset of tics between individuals with GTS alone and those with GTS and ASD. The incidence of GTS and ASD comorbidity in this study was high, and this has several implications in terms of treatment and prognosis.

## 1. Introduction

Gilles de la Tourette syndrome (GTS) is a neurodevelopmental disorder characterized by the presence of motor tics and at least one vocal tic that occurs for more than one year, with an onset age before 18 years (APA) [1]. Autism spectrum disorder (ASD) is also a neurodevelopmental disease, characterized by persistent deficits in social communication and social interaction across multiple contexts, together with restricted and repetitive patterns of behavior and interests or activities (APA). The incidence of GTS alone in an individual is rare; it is more frequently associated with comorbidities that have a greater effect than tics on the daily life of the patient [2,3]. Several comorbidities are reported in association with GTS, such as obsessive–compulsive disorder (OCD), attention deficit hyperactivity disorder (ADHD), anxiety [4], depression [5], and ASD [6,7,8,9,10]. The incidence and the concomitant presence of OCD, ADHD, and mood disorders have been studied in depth in recent years [3,11]. As far as we know, the incidence of GTS and comorbid ASD has not been studied as extensively. This could be because the phenomenology of these comorbidities is not well known.

GTS and ASD are etiologically related; studies on GTS in ASD have reported incidences between 2.9% and 20% [6,7,8,9,10]. The prevalence of ASD alone is significantly lower: 1.9% in children aged 0–15 years and 1.2% in young adolescents aged 4–16 years [12]. Several studies have reported a common genetic basis for these disorders, such as deletions and microduplications [13,14,15,16,17]. Moreover, the Brainstorm Consortium presented evidence of the shared heritability in both disorders [18].

The aim of the present study was to identify the incidence of ASD in a large clinical sample of GTS patients recruited from the Catania Tourette Clinic and to compare our data with the incidences reported in previous studies.

## 2. Materials and Methods

### 2.1. Patient Selection

We retrospectively analyzed the clinical data obtained between January 2010 and March 2019 from the GTS database of the outpatient Catania Tourette Clinic, a section of the Child and Adolescent Neurology and Psychiatry of the Medical and Experimental Department of Catania University. Out of 1200 cases, the data of 975 affected individuals of various socioeconomic statuses were included in this study. The diagnoses of GTS and ASD were made according to the diagnostic and statistical manual of mental disorders (DSM 5 and DSM-IV-TR) criteria and neuropsychological evaluation was carried out by an expert and a certificated team of child and adolescent neurologists, supervised by two expert child neurologists (R.R. and M.G.). To measure the inter-rater reliability we used the Fleiss Kappa coefficients between raters results and it was 0.87 (strong level of agreement and reliability of results about 80%) [19].

We enrolled pharmacologically naïve patients with normal intelligence quotient (IQ) levels (total IQ score = 100 ± 2), namely, those with a borderline IQ (total IQ score = 75–70) and those with mild mental retardation (total IQ score = 55–70) who were able to understand and complete the evaluation. We excluded all patients who showed evidence of severe neurological or physical impairment/disease with comorbidities, patients who were unable to communicate, and patients with chronic and provisional tic disorders. Initially, we analyzed the complete data, after which, we performed an age-stratified analysis with two subgroups: 5–11 years and 12–18 years. We decided to perform the analysis with age stratification to catch differences between ages because of the differences in the comorbidities reported in GTS patients at different ages. The data included were without the influence of drugs or behavioral therapy, collected at the beginning, just after the completion of the diagnostic process.

### 2.2. Ethics and Procedures

This study was approved by the local ethics committee (Catania 1). All parents provided written informed consent, although the subjects assented when possible. All patients underwent physical examination and an ECG, and blood and urine samples were collected. With regard to the blood and urine samples, we performed them:

(i) To verify if patients met the inclusion/exclusion criteria, because sometimes, tics can be a symptom of another disease; with this in mind, we analyzed ceruloplasmin to exclude Wilson’s disease, thyroid hormones to exclude hyperthyroidism, amino acids to exclude metabolic disease, and a peripheral blood smear to exclude neuroacanthocytosis;
(ii) All other levels (e.g., blood count, glycemia, azotemia, creatinine, prolactin, antistreptolysin o titre and throat swab, and transaminases) and urine samples were checked, and an ECG was performed, in order to gain a general picture of the patient’s state of health, which could be useful in case the patient needed pharmacological treatment.

Additionally, all patients underwent a complete neuropsychological evaluation.

### 2.3. Assessments

All the patients were screened using the Schedule for Affective Disorders and Schizophrenia for School-Age Children—Present and Lifetime version (K-SADS-PL) [20] in order to diagnose comorbid psychiatric disorders that may have excluded them from the study. The intelligence quotient was calculated using the Wechsler Scales [21], and parents also completed a semi-structured interview, namely, the Vineland Adaptive Scale [22], to determine the correct level of impairment on the basis of adaptative functions, as suggested by the DSM-5 [1]. GTS was diagnosed using a semi-structured interview, namely, the National Hospital Interview Schedule (NHIS) for GTS [23]. In addition, the Yale Global Tic Severity Rating Scale (YGTSS) [24] was used to assess the severity of GTS. Patients diagnosed with GTS underwent a neuropsychological evaluation for the verification of comorbidities. The Children Yale Brown Obsessive–Compulsive Scale (CY-BOCS) was used to evaluate the presence and severity of OCD [25]. The DSM-IV–5 criteria were used for ADHD diagnosis [1]. Finally, ASD was diagnosed using the Autism Diagnostic Observation Schedule (ADOS) [26] for the children, while parents completed the Autism Diagnostic Interview—Revised (ADI-R) structured interview [27].

### 2.4. Statistical Analysis

Student’s *t*-tests and one-way/two-way analyses of variance (ANOVAs) were used to compare the neuropsychological scores and characteristics between groups. A *p*-value ≤ 0.05 was considered to indicate statistical significance.

## 3. Results

### 3.1. Sample Characteristics

The characteristics of the participants are summarized in Table 1.

In this study, we included 975 individuals diagnosed with GTS aged 5–18 years (mean age = 12.4 ± 6.4; male (M)/female (F) = 813:162; male = 81%). The mean age of tic onset was 6.4 years (±2.4), while the mean age of the first visit was 12.4 years (±6.4).

**Figure 1:**
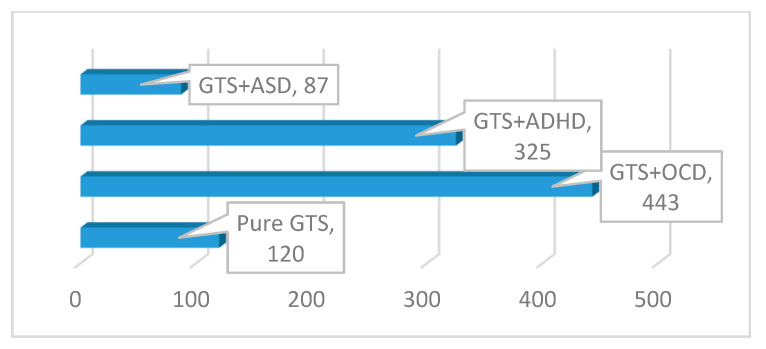
The GTS clinical subgroups. GTS: Gilles de la Tourette syndrome; ASD: Autism spectrum disorder; OCD: Obsessive–compulsive disorder; ADHD: Attention deficit hyperactivity disorder.

The identified clinical subgroups were as follows: GTS only (2.3%; *n* = 120), GTS + OCD (45.5%; *n* = 443), GTS + ADHD (33.3%; *n* = 325), and GTS + ASD (8.9%; *n* = 89) (Table 1 and Figure 1).

Moreover, we identified GTS sub-phenotypes as follows: GTS only, GTS + ADHD, GTS + OCD, and GTS + ASD. We stratified our sample into two groups based on age, as follows: (i) 526 children aged 5–11 years (mean age = 8.7 ± 1.6; M/F = 453:73), and (ii) 446 adolescents aged 12–18 years (mean age = 14.1 ± 1.3; M/F = 360:89). With regard to blood, urine, and ECG analyses, all of the patients included in the study presented normal values.

### 3.2. Incidence of ASD in GTS

Among the individuals diagnosed with GTS, 8.9% (*n* = 87) also had ASD. The incidence of ASD in GTS was statistically significant in the group of children compared to the group of adolescents (*p* < 0.001). Specifically, 2.6% (*n* = 14) children presented ASD in GTS compared to 17% (*n* = 76) of the adolescents. The M/F ratio was 12:2 (male = 85%) in the group of children (*n* = 14) compared to 64:12 (male = 84%) in the group of adolescents. No statistically significant differences were observed based on sex distribution or based on the age of onset of tics in the GTS only versus the GTS + ASD group (*p* = 0.2345). Moreover, no differences were found in the age-stratified subgroups either.

### 3.3. Neuropsychological Evaluation

The results of the neuropsychological evaluation are summarized in Table 2.

**Figure 2:**
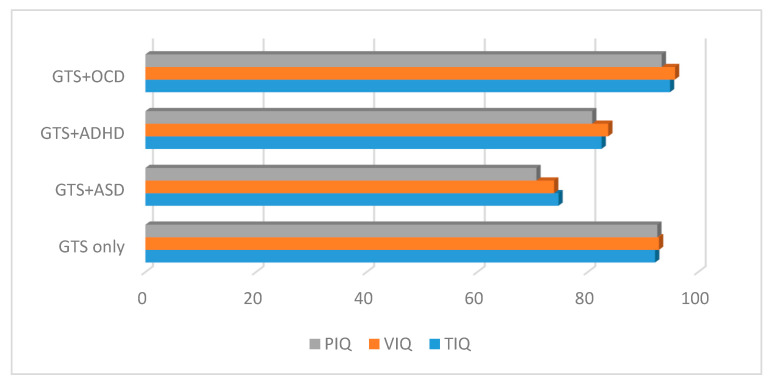
IQs in the GTS clinical subgroups. GTS: Gilles de la Tourette syndrome; ASD: Autism spectrum disorder; OCD: Obsessive–compulsive disorder; ADHD: Attention deficit hyperactivity disorder; IQ: Intelligence quotient; PIQ: Performance intelligence quotient; VIQ: Verbal intelligence quotient; TIQ: Total intelligence quotient.

The IQs of the patients in the GTS + ASD subgroup were statistically significantly lower than those in the GTS only, GTS + ADHD, and GTS + OCD subgroups (*p* = 0.000) (see Table 2 and Figure 2).

These differences were apparent in the complete cohort analysis, as well as in the age-stratified analysis.

**Figure 3:**
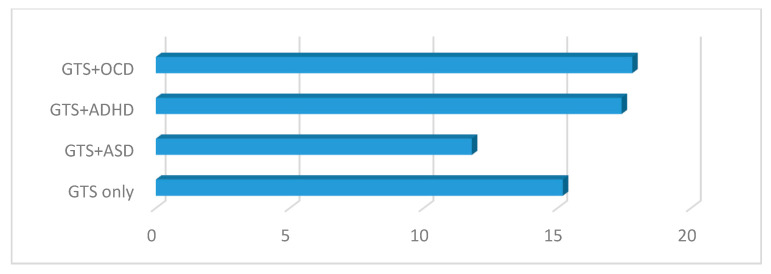
YGTSS in the GTS clinical subgroups. GTS: Gilles de la Tourette syndrome; ASD: Autism spectrum disorder; OCD: Obsessive–compulsive disorder; ADHD: Attention deficit hyperactivity disorder; YGTSS: Yale Global Tic Severity Scale.

Statistically significant differences were observed between the GTS subgroup scores (GTS only, GTS + ASD, GTS + OCD, and GTS + ADHD) in the severity of tics as assessed by YGTSS (*p* < 0.000). Similar results (*p* < 0.000) were obtained in the age-stratified groups as well. Importantly, the severity of tics was worse when comorbid ADHD and OCD were present (GTS + OCD vs. GTS + ADHD, *p* = 0.412; *t* = 0.820) (see Table 2 and Figure 3).

**Figure 4:**
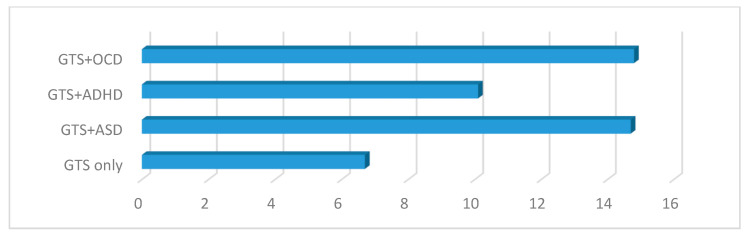
CY-BOCS in the GTS clinical subgroups. GTS: Gilles de la Tourette syndrome; ASD: Autism spectrum disorder; OCD: Obsessive–compulsive disorder; ADHD: Attention deficit hyperactivity disorder; CY-BOCS: Children Yale Brown Obsessive–Compulsive Scale.

Statistically significant differences were also observed in the CY-BOCS scores between the GTS subgroups (GTS only, GTS +ASD, GTS + OCD, and GTS + ADHD) (*p* < 0.000) (see Table 2 and Figure 4).

In particular, GTS patients with comorbid OCD and ASD presented non-statistically significant different scores between one another (GTS + ASD vs. GTS + OCD, *p* = 0.910; *t* = 0.112), and statistically significantly higher scores than the GTS only and GTS + ADHD subgroups (see Table 3). Similar results were obtained in the age-stratified analysis.

**Figure 5:**
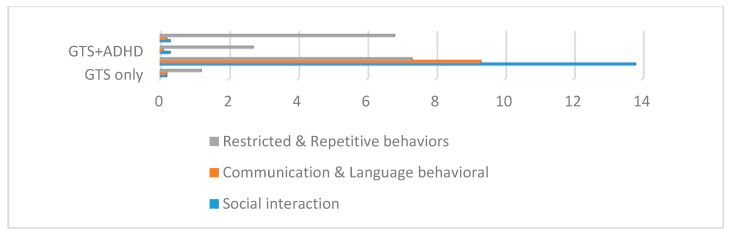
ADI-R in the GTS clinical subgroups. GTS: Gilles de la Tourette syndrome; ASD: Autism spectrum disorder; OCD: Obsessive–compulsive disorder; ADHD: Attention deficit hyperactivity disorder; ADI-R: Autism Diagnostic Interview—Revised.

**Figure 6:**
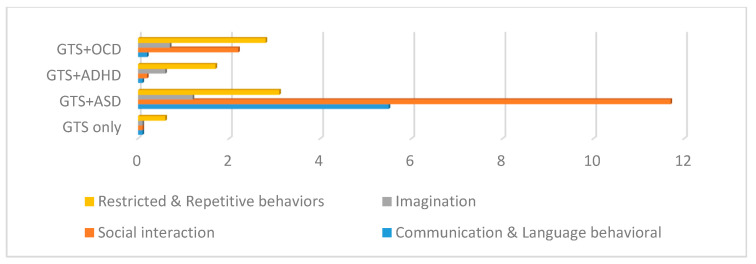
ADOS in the GTS clinical subgroups. GTS: Gilles de la Tourette syndrome; ASD: Autism spectrum disorder; OCD: Obsessive–compulsive disorder; ADHD: Attention deficit hyperactivity disorder; ADOS: Autism Diagnostic Observation Schedule.

All of the patients of the GTS and ASD group fulfilled all of the criteria for a diagnosis using both the ADOS and ADI-R scales. The comparison between the mean ADOS and ADI-R scores in the GTS clinical subgroups showed statistically significant differences (*p* < 0.000). In detail, the GTS only and GTS + ADHD subgroups presented statistically significant scores in all fields of the ADOS and ADI-R; in contrast, only in the “restricted and repetitive behaviors” measure were no statistically significant differences found in either ADOS or the ADI-R (see Table 4 and Figure 5 and Figure 6).

GTS: Gilles de la Tourette syndrome; ASD: Autism spectrum disorder; OCD: Obsessive–compulsive disorder; ADOS: Autism Diagnostic Observation Schedule; ADI-R: Autism Diagnostic Interview—Revised.

## 4. Discussion

In this clinical retrospective study, we analyzed a large clinical sample of patients with GTS who were diagnosed in a single Italian Tourette clinic, in order to identify the incidence of ASD in GTS. We also briefly analyzed the results of each GTS clinical group.

We included 975 individuals diagnosed with GTS aged 5–18 years (mean age = 12.4 ± 6.4; M/F = 813:162; male = 81%) and found that 12.3% had GTS only, 33.3% had GTS + ADHD, 45.5% had GTS + OCD, and 8.9% had GTS + ASD. We found statistically significant differences in IQ, especially between the GTS only and GTS + ASD subgroups. Our data are in line with the recent literature, in which intellectual disability is associated with ASD in approximately 75% of patients [28].

Concerning the neuropsychological findings:

(i) The YGTSS scores are in line with previous studies [2,29] stating that high incidence of OCD and ADHD in GTS can cause poor quality of life and distress, and worsen symptoms the symptoms of GTS with higher YGTSS scores [30]. On the contrary, we did not find a higher YGTSS score in patients with GTS + ASD. This could be explained by the lower mean IQ, which could have resulted in a lower consciousness [31];
(ii) The CY-BOCS scores showed statistically significant differences between all four GTS subgroups. Patients with GTS + OCD and GTS + ASD presented higher scores, but they were not statistically different compared to those of the GTS only and GTS + ADHD subgroups. These results are of interest, as repetitive behaviors are observed in as many as 65% of patients with GTS and can be classified as “tic-like” or OCD-like symptoms according to the clinical phenomenology [30]. Repetitive behaviors in ASD typically overlap with the phenomena in GTS; however, it may be challenging to distinguish the phenomenological characteristics of ASD from those of GTS. In clinical practice, medical professionals often find it difficult to define the disorder that best describes a child’s symptoms [31];
(iii) Finally, we found that among the individuals diagnosed with GTS, 8.9% (*n* = 87) also had ASD. The data reported in literature have reported a wide range, between 2.9% to 20% [6,7,8,9,10].

In this section, we attempt to discuss our results in light of previous studies. First, it is necessary to specify that studies conducted on the general population could be affected by Berkson’s bias. Kadesjö and Gilberg [32] found that GTS is present in 0.15–1.1% of the general population, with males outnumbering females by 4:1 through 6:1. They found that 9% of ASD patients had GTS, using the Conner’s scale as a screening instrument.

Khalifa and von Knorring (2006) [33], using the Autism Spectrum Screening Questionnaire, found a GTS incidence of 0.6% in the general population, in which the rate of ASD was 20%. In our study, we found that in the 975 patients with GTS, 8.9% presented comorbid ASD. To verify the concordance of the data, we reviewed the literature in light of our results and found a limited number of studies that reported the incidence of ASD in GTS in the clinical population.

In 2009, Burd et al. [6] conducted a clinical cohort study using the data from the Tourette Syndrome International Consortium Registry (*n* = 7288). They found that 334 patients (4.6%) fulfilled the criteria of the DSM-IV for ASD. This incidence is lower than ours; however, this study presented two limitations that could interfere with the results: (i) No specific neuropsychological evaluation instruments were used for the diagnosis of ASD, and (ii) the age of the sample was not specified.

Ghanizadeh et al. (2009) [8] conducted a clinical study in Iran on 35 children and adolescents aged 6–18 years (mean age = 11.8 years) who were referred to the Service of Child and Adolescent Psychiatry of the Shiraz Medical Hospital. All subjects underwent neuropsychological evaluation with the following schedules and criteria: K-SADS-PL, YGTSS, the Child Behavior Check List, and the DSM-IV criteria. In this sample, they found that GTS alone was rare and that GTS with comorbid ASD had an incidence of 2.9% (*n* = 1). However, the study was conducted in a very small sample that was most likely not representative of all the affected patients, as (i) ASD was diagnosed only with the DSM-IV criteria and (ii) a unique group of children and adolescents aged 6–18 years was included without specifying the number of children and adolescents. Therefore, this could be a confounding factor because the phenomenology of both disorders is very different at different ages.

Huisman-van Dijk et al. (2016) [9] conducted a clinical cohort study on 225 patients with GTS aged 6–72 years who were recruited from two specialized Dutch outpatient clinics. They were assessed using the YGTSS, Child Behavior Check List (CBCL), Conner’s scale, Autism Spectrum Quotient, and SCID-I. The researchers were able to identify patients who met the criteria for probable ASD, and concluded that 45 patients (20%) presented possible comorbid ASD. This is higher than the incidence observed by us; in our opinion, two factors could have interfered with this data. First, they did not use a gold standard evaluation scale for the diagnosis of ASD and, thus, only reported “possible ASD”. Second, the age range that they considered was comparatively wide.

Darrow et al. (2017) [7] recruited 535 patients with GTS, older than 6 years (mean age = 30.9 ± 18.2), from the Tourette Syndrome Association Consortium for Genetics. All of the patients were interviewed for ASD twice, in person or via Skype or telephone. They used the second edition of the Social Responsiveness Scale (SRS-II) to characterize ASD symptoms and found an incidence of probable ASD of 22.8% in children and adolescents, and 8.7% in adults. The major limitation of this study was the unique use of SRS-II, because, as the authors themselves stated in their paper, this scale is not specific for ASD, and its score only reflects psychiatric impairment. They concluded that, in order to confirm these incidences, ADOS and ADI-R should be used.

Cravedi et al. [10] conducted a clinical study to explore the heterogeneity of GTS through hierarchical ascendant clustering analysis. They included 174 GTS children and adolescents aged <17 years (mean age = 11.4 ± 3.4). To diagnose ASD, suspected patients were evaluated using the Autism Mental Status Exam (AMSE), a clinical observational assessment scale composed of eight items, to detect ASD symptoms; parents of all patients who presented a score of 5 points or higher subsequently completed the ADI-R to confirm the diagnosis, and ASD was confirmed in 24 (14%) of the patients. The higher incidence found by Cravedi et al. (14% vs. 8.9%) may be related to the cut-off of AMSE that was used to identify ASD children. Recently, Cederlund (2019) reported that AMSE is a valid instrument, but the optimal cut-off score for a probable diagnosis of ASD, according to ICD10 criteria, is 7 with a sensitivity and specificity of 75% and 78%, respectively [34].

A probable genetic cause could be responsible for both the disorder and the increasing incidence. Yang et al. (2019) [35] conducted a systematic cross-disorder meta-analysis, using available GWAS data to find the genetic overlap between ADHD, GTS, OCD, and ASD. First of all, they found, using cross-disorder tissue-specific analysis, that the hypothalamus–pituitary–adrenal axis is possibly implicated in the pathophysiology of ASD, ADHD, GTS, and ASD; thus, all of these conditions are strictly related to stress. With regard to GTS and ASD, they analyzed a dataset of 7,499,503 single-nucleotide polymorphisms (SNPs); only 15 SNPs were genome-wide significant for both disorders, and four independent risk regions were identified. It is probable that these SNPs are involved in the pathophysiology of these disorders because they interfere with the development of some interneurons and are responsible for the involvement of some pathways related to neuronal development, axogenesis, synaptic structures, and organization [36]. However, further research is needed.

## 5. Conclusions

We believe that this is the first single-site study on a large cohort of patients with GTS in which a complete assessment for GTS and comorbidities was performed using the gold standard clinical and neuropsychological evaluation tools (i.e., YGTSS, CY-BOCS, ADOS, and ADI-R). We found that 8.9% of the patients diagnosed with GTS also presented comorbid ASD and that the majority of the patients affected were children. These data are important due to the implications in the clinical field, as 8.9% is a quite high percentage of patients. In our opinion, it is important that clinicians keep in mind that, sometimes, the restrictive and repetitive behaviors that GTS patients present are not related to OCD but instead to ASD. As per a recent paper of ours [37], specific instruments (e.g., CY-BOCS for ASD) could be used to discriminate ASD symptoms from OCD symptoms, and, of course, this could improve the diagnosis and, consequently, inform the correct treatment. Additionally, antipsychotics are not as effective in this case compared to other GTS cases. Further research is required to explain all of the differences observed and to improve the quality of life and social aspects of this subgroup of patients with GTS.

## Figures and Tables

**Table 1:** Demographic and clinical features of the participants.

	GTS (*n* = 975)
Male (M)/female (F)	813:162
Age of onset (tic)	6.4 ± 2.4
Mean age	12.4 ± 6.4
Clinical subgroups (%)	
Pure GTS	12.3 (*n* = 120)
GTS + ASD	8.9 (*n* = 87)
GTS + OCD	45.5 (*n* = 443)
GTS + ADHD	33.3 (*n* = 325)

GTS: Gilles de la Tourette syndrome; ASD: Autism spectrum disorder; OCD: Obsessive–compulsive disorder; ADHD: Attention deficit hyperactivity disorder; IQ: Intelligence quotient; YGTSS: Yale Global Tic Severity Scale; CY-BOCS: Children Yale Brown Obsessive–Compulsive Scale.

**Table 2:** Neuropsychological findings of the GTS patients.

Measures	GTS Only	GTS + ASD	GTS + ADHD	GTS + OCD
IQ				
TIQ	92.2 (17.8)	74.7 (14.8)	82.5 (9.2)	94.9 (17.6)
VIQ	92.9 (18.6)	73.9 (12.5)	83.7 (10.7)	95.8 (18.5)
PIQ	92.6 (7.5)	70.73 (9.4)	80.8 (8.7)	93.4 (19.1)
YGTSS	15.2 (7.6)	11.8 (2.3)	17.4 (6.9)	17.8 (6.5)
CY-BOCS	6.7 (6.1)	14.7 (9.8)	10.1 (6.8)	14.8 (7.1)
ADI-R				
Social interaction	0.2 (0.9)	13.8 (6.8)	0.3 (0.8)	0.3 (0.9)
Communication and language behavioral	0.2 (0.6)	9.3 (5.1)	0.1 (0.2)	0.2 (0.6)
Restricted and repetitive behaviors	1.2 (2.2)	7.3 (3.6)	2.7 (3.7)	6.8 (3.1)
ADOS				
Communication and language behavioral	0.1 (0.3)	5.5 (1.2)	0.1 (0.2)	0.2 (0.4)
Social interaction	0.1 (0.6)	11.7 (5.6)	0.2 (0.2)	2.2 (0.7)
Imagination	0.1 (0.1)	1.2 (1.9)	0.6 (0.2)	0.7 (0.4)
Restricted and repetitive behaviors	0.6 (1.1)	3.1 (2.2)	1.7 (1.5)	2.8(1.3)

GTS: Gilles de la Tourette syndrome; ASD: Autism spectrum disorder; OCD: Obsessive–compulsive disorder; ADHD: Attention deficit hyperactivity disorder; IQ: Intelligence quotient; PIQ: Performance intelligence quotient; VIQ: Verbal intelligence quotient; TIQ: Total intelligence quotient; YGTSS: Yale Global Tic Severity Scale; CY-BOCS: Children Yale Brown Obsessive–Compulsive Scale; ADOS: Autism Diagnostic Observation Schedule; ADI-R: Autism Diagnostic Interview—Revised.

**Table 3:** Comparison of the CY-BOCS scores between GTS subgroups.

	*p-*Value	*t*-Value
GTS + ASD vs. GTS only	0.000	7.221
GTS + ASD vs. GTS + ADHD	0.000	5.061
GTS + ASD vs. GTS + OCD	0.910	0.112
GTS only vs. GTS + ADHD	0.000	4.808
GTS only vs. GTS + OCD	0.000	11.407
GTS + OCD vs. GTS + ADHD	0.000	9.226

GTS: Gilles de la Tourette syndrome; ASD: Autism spectrum disorder; OCD: Obsessive–compulsive disorder; ADHD: Attention deficit hyperactivity disorder.

**Table 4:** Comparison of the ADOS and ADI-R “repetitive and restricted behaviors” scores between the GTS + ASD and GTS + OCD subgroups.

	*p-*Value	*t*-Value
**ADOS**	0.085	1.723
**ADI-R**	0.181	1.337
